# Impact of Regular Physical Activity on Aortic Diameter Progression in Paediatric Patients with Bicuspid Aortic Valve

**DOI:** 10.1007/s00246-021-02591-4

**Published:** 2021-04-16

**Authors:** Emanuele Monda, Adelaide Fusco, Alessandro Della Corte, Martina Caiazza, Annapaola Cirillo, Felice Gragnano, Maria Pina Giugliano, Rodolfo Citro, Marta Rubino, Augusto Esposito, Arturo Cesaro, Francesco Di Fraia, Giuseppe Palmiero, Marco Di Maio, Marcellino Monda, Paolo Calabrò, Giulia Frisso, Stefano Nistri, Bicuspid Aortic Valve Consortium (BAVCon), Eduardo Bossone, Simon C. Body, Maria Giovanna Russo, Giuseppe Limongelli

**Affiliations:** 1 Department of Translational Medical Sciences, University of Campania “Luigi Vanvitelli”, Naples, Italy; 2 Division of Cardiology, A.O.R.N. Sant’Anna e San Sebastiano, Caserta, Italy; 3 Cardiovascular Department, University Hospital “San Giovanni di Dio e Ruggid’Aragona”, Salerno, Italy; 4 Department of Cardiology, AO deiColli, Monaldi Hospital, Naples, Italy; 5 Department of Experimental Medicine, Section of Human Physiology and Unit of Dietetics and Sports Medicine, University of Campania “Luigi Vanvitelli”, Naples, Italy; 6 Department of Molecular Medicine and Medical Biotechnology, Federico II University of Naples, Naples, Italy; 7 Cardiology Service, CMSR Veneto Medica, Altavilla Vicentina, Italy; 8 Division of Cardiology, Antonio Cardarelli Hospital, Naples, Italy; 9 Department of Anesthesiology, Boston University School of Medicine, Boston, MA USA; 10 Institute of Cardiovascular Sciences, University College of London and St. Bartholomew’s Hospital, London, UK

**Keywords:** Bicuspid aortic valve, Aortopathy, Echocardiography, Paediatrics, Exercise

## Abstract

Patients with bicuspid aortic valve (BAV) have an increased risk of aortic dilation and aortic dissection or rupture. The impact of physical training on the natural course of aortopathy in BAV patients remains unclear. The aim of this study was to evaluate the impact of regular physical activity on aortic diameters in a consecutive cohort of paediatric patients with BAV. Consecutive paediatric BAV patients were evaluated and categorized into two groups: physically active and sedentary subjects. Only the subjects with a complete 2-year follow-up were included in the study. To evaluate the potential impact of physical activity on aortic size, aortic diameters were measured at the sinus of Valsalva and mid-ascending aorta using echocardiography. We defined aortic diameter progression the increase of aortic diameter ≥ 10% from baseline. Among 90 BAV patients (11.5 ± 3.4 years of age, 77% males), 53 (59%) were physically active subjects. Compared to sedentary, physically active subjects were not significantly more likely to have > 10% increase in sinus of Valsalva (13% vs. 8%, *p*-value = 0.45) or mid-ascending aorta diameter (9% vs. 13%, *p*-value = 0.55) at 2 years follow-up, both in subjects with sinus of Valsalva diameter progression (3.7 ± 1.0 mm *vs.* 3.5 ± 0.8 mm, *p*-value = 0.67) and in those with ascending aorta diameter progression (3.0 ± 0.8 mm *vs*. 3.2 ± 1.3 mm, *p*-value = 0.83). In our paediatric cohort of BAV patients, the prevalence and the degree of aortic diameter progression was not significantly different between physically active and sedentary subjects, suggesting that aortic dilation is unrelated to regular physical activity over a 2-year period.

## Introduction

Bicuspid aortic valve (BAV) is the most common congenital valvular heart abnormality, affecting 1–2% of the general population [1]. BAV patients have an increased risk of developing infective endocarditis, aortic stenosis, aortic regurgitation and progressive aortic dilation [2–4]. The aortic dilation associated with BAV may be a risk factor for aortic dissection or rupture [5, 6] and is generally disproportionate to the associated valvular lesion [7]. In these patients, aortic dilation has been explained by histological abnormalities of the ascending aorta [8].

The importance of pre-participation screening in athletes is based on the supposition that intense athletic training and competitive activity can be a mechanism of increased risk of aortic dilation [9]. The AHA/ACC Task Force 7 recommends frequent follow-up of BAV patients with a mild to moderately dilated aorta and prudentially refraining from competitive sports in those with a moderate to severely dilated aorta [10]. Nevertheless, though the diagnosis of BAV in subjects with normal aortic diameter and valve function after pre-participation screening is not felt to be a limitation for sport activities, inappropriate restrictions and disqualification of BAV patients with mild-to-moderate dilatation from sport activities is frequently encountered in clinical practice.

The impact of physical training and competitive sports on the natural course of aortopathy in patients with BAV is not fully known, particularly in children [11]. It is not known if physiological stress associated with regular and intense physical activity may favour valve deterioration or aortic root and ascending aorta dilation.

The aim of this study was to evaluate the impact of regular physical activity on aortic diameters in a consecutive cohort of paediatric patients with BAV.

## Material and Methods

### Study Population and Definitions

Consecutive paediatric patients with isolated BAV were prospectively evaluated between January 2016 and January 2018 at the Inherited and Rare Cardiovascular Diseases Clinic of the University of Campania “Luigi Vanvitelli”, Naples, Italy. All patients with a genetic syndrome and/or with complex congenital heart diseases were excluded.

BAV was defined as a congenital bicuspid aortic valve disease comprising a spectrum of deformed aortic valves presenting with two functional cusps forming a valve mechanism with less than three zones of parallel apposition between cusps [12].

The patients were categorized into two groups: physically active and sedentary subjects. The physically active subjects' group was composed by individuals who practiced regular leisure-time physical activity at least three times a week for at least 10 months a year for the entire follow-up period. The sedentary subjects' group was composed of individuals that did not practice regular physical activity. Among the BAV patients examined, only subjects with a complete 2-year follow-up were included in the study.

### Study Protocol

Patients were enrolled after informed consent was obtained, according to the procedure established by the Ethics Committee of our institution. All patients underwent a comprehensive evaluation, including pedigree, medical history, standard 12-lead ECG, general laboratory investigation, conventional M-mode, two-dimensional, Doppler echocardiography and Doppler tissue imaging, 24-h ECG Holter, and when required, cardiac magnetic resonance imaging (CMR). Clinical evaluation including standard ECG and echocardiography was repeated every 6 months and laboratory evaluation and ECG monitoring were performed at least once a year.

### Echocardiography

**Fig. 1:**
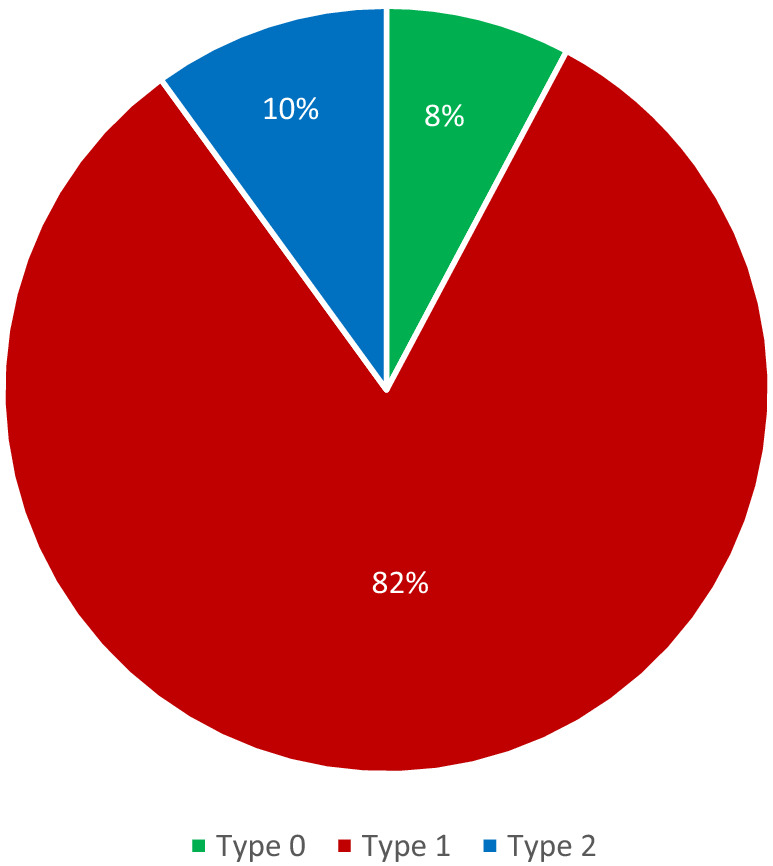
Distribution of bicuspid aortic valve morphologies in our cohort. Type 0, valves with no raphe; type 1, valves with one raphe; type 2, valves with two raphes

The diagnosis of BAV was confirmed when two cusps and two commissures were clearly identified in systole and diastole in the short-axis view. BAV was classified according to the number of fibrous raphes, codifying the BAVs into three types: type 0, valves with no raphe; type 1, valves with one raphe; and type 2, valves with two raphes [12]. In type 1, sub-classes of cusp fusion were arbitrated (Fig. 1).

In order to evaluate the potential impact of the physical activity on the aortic root measurement, its diameters were measured at two levels (sinus of Valsalva and ascending aorta) in the parasternal long-axis view. According to the current recommendations [13], aortic root measurements were made at end-diastole, in a perpendicular plane to that of the long axis of the aorta using the L–L convention. A z score was also calculated for each aortic measurement [14]. Sinus of Valsalva or ascending aorta dilation was defined as a *z* score ≥ 2 and was classified as mild (*z* score ≥ 2 and ≤ 4), or severe (*z* score > 4).

We defined aortic diameter progression as an increased sinus of Valsalva or ascending aorta diameter ≥ 10% from baseline, associated with a z score increase, to take into account the growth rate of aortic diameter and to minimize potential bias related to inter- and intra-observer variability of echocardiographic aortic measurements.

### Statistical Analysis

Data are presented as percentages, means and standard deviations. Normally distributed continuous data are presented as mean ± standard deviation (SD) and were compared by *t*-test. Categorical variables were expressed as number (percentage) and analysed by Chi-square test or Fisher exact test, where appropriate. *p*-values < 0.05 (two-tailed) were considered significant. All statistical analyses were performed using SPSS (version 15.0, 2002, SPSS Inc., Chicago, Illinois, USA).

## Results

### General Characteristics

The BAV population examined was composed of 90 patients, including 53 physically active subjects (59% of the entire population). Clinical characteristics of the population are reported in Table 1. The mean age at study enrollment of the total population was 11.5 ± 3.4 years old, 77% were males and 75% patients were diagnosed using 2D echocardiography for a cardiac murmur at physical examination. The most common BAV class found at echocardiography was BAV type 1 with a right-left cusp fusion (Table 1).

**Table 1:** Clinical characteristics of the examined cohort

Clinical features	Total population (*n* = 90)
Age at diagnosis, years	4.1 ± 4.4
Age at study enrolment, years	11.6 ± 3.4
Body surface area, m^2^	1.4 ± 0.4
Males	67 (76.7)
Diagnosis	
Incidental	14 (15.5)
Cardiac murmur	68 (75.5)
Symptomatology	8 (8.9)
Physically active subjects	53 (58.9)

Data are presented in mean ± SD or *n* (%)

### Physically Active Subjects' Group

Physically active subjects with BAV participated in several sports: 23% swimming, 22% track and field athletics, 19% soccer, 13% martial arts, 13% dancing, and the remaining 10% practiced other sports such as basketball and volleyball. BAV type 1 was the most common variant found at echocardiography, present in 44 (83%), and R–L morphology was the most common subtype in these subjects (70% of BAV type 1 patients) (Table 2).

**Table 2:** Clinical characteristics of the two groups

Clinical features	Physically active subjects' group (*n* = 53)	Sedentary subject' group (*n* = 37)	*p*-value
Age at diagnosis, years	4.8 ± 4.7	3.2 ± 3.7	0.088
Age at study enrolment, years	13.2 ± 2.5	9.3 ± 3.0	< 0.001*
Body surface area, m^2^	1.6 ± 0.3	1.3 ± 0.3	< 0.001*
BAV morphology			0.970
Type 0	4 (7.5)	3 (8.1)	0.922
Type 1	44 (83)	30 (81.1)	0.813
Subtype R-L	31 (58.5)	21 (56.7)	0.966
Subtype R-NC	13 (24.5)	9 (24.3)	0.966
Type 2	5 (9.4)	4 (10.8)	0.830
Aortic regurgitation	28 (52.8)	19 (51.3)	0.890
Mild	24 (45.3)	11 (29.7)	0.136
Moderate	3 (5.7)	5 (13.5)	0.198
Severe	1 (1.9)	3 (8.1)	0.159
Sinuses of Valsalva diameter mm	27.2 ± 4.3	23.8 ± 3.9	< 0.001*
z score, median (IQR)	0.7 (1.9)	0.4 (1.6)	0.253
Sinuses of Valsalva dilation, mild (≥ 2 and ≤ 4 *z* score) severe (> 4 * z* score )	8 (15.1)	5 (13.5)	0.834
	8 (15.1)	4 (10.8)	0.556
	0 (0)	1 (2.7)	0.229
Ascending aorta diameter mm	28.3 ± 5.1	25.6 ± 4.4	0.009*
z score, median (IQR)	2.4 (2.5)	2.8 (2.6)	0.633
Ascending aorta dilation, mild (≥ 2 and ≤ 4 * z* score ) severe (> 4 * z* score )	33 (62.3)	21 (56.7)	0.600
	20 (37.7)	16 (43.2)	0.600
	13 (24.5)	5 (13.5)	0.199
Sinus of Valsalva diameter progression > 10 mm	7 (13.2)	3 (8.1)	0.449
Ascending aorta diameter progression > 10 mm	5 (9.4)	5 (13.5)	0.545

Data are presented in mean ± SD or *n* (%), unless otherwise indicated

*BAV* bicuspid aortic valve

*p-values < 0.05 were considered statistically significant

With colour Doppler echocardiography, the most prevalent valve dysfunction found in physically active subjects was aortic regurgitation (AR), present in 28 individuals (53%) with severity of mild, moderate and severe in 45%, 6% and 2%, respectively. During 2-year follow-up, 7 (13%) physically active subjects showed sinus of Valsalva diameter progression and 5 (9%) showed ascending aorta diameter progression (Table 2).

### Sedentary Subjects' Group

The sedentary group was composed of 37 subjects (41% of the entire BAV population). The most common BAV morphology was type 1, present in 30 subjects (81%), and subtype R-L was identified in 70% of them, while the R-NC was found in 30% (Table 2). AR was found in 19 sedentary subjects (51%) and the degree of AR was mild, moderate and severe in 30%, 13% and 8%, respectively. During 2-year follow-up, 3 (8%) sedentary subjects showed sinus of Valsalva diameter progression and 5 (13%) showed ascending aorta diameter progression (Table 2).

### Impact of Physical Activity in Patients with BAV

Clinical characteristics of the examined cohort, including mean age at diagnosis, BAV type and subtype, presence and degree of AR, aortic sinus and ascending aorta z score or dilation did not significantly differ between the two groups (Table 2). Compared to baseline, no significant difference in sinus of Valsalva or ascending aorta diameter was observed at 2-year follow-up, in either group (Table 3). Also, at 2 years follow-up, no significant difference in aortic diameter changes was reported between the two groups (Table 4).

**Table 3:** Echocardiographic findings of study population at baseline and at 2 years follow-up

Clinical features	Baseline	2 years follow-up	*p*-value
Physically active subjects' group			
Age at study enrolment, years	13.2 ± 2.5	15.2 ± 2.5	< 0.001*
Sinuses of Valsalva diameter mm	27.2 ± 4.3	27.3 ± 4.5	0.863
z score, median (IQR)	0.7 (1.9)	0.6 (1.9)	0.212
Ascending aorta diameter mm	28.3 ± 5.1	28.4 ± 5.3	0.733
z score, median (IQR)	2.4 (2.5)	2.5 (2.7)	0.354
Sedentary subjects' group			
Age at presentation, years	9.3 ± 3.0	11.3 ± 3.0	< 0.001*
Sinuses of Valsalva diameter mm	23.8 ± 3.9	24.5 ± 4.4	< 0.001*
z score, median (IQR)	0.4 (1.6)	0.6 (1.3)	0.174
Ascending aorta diameter mm	25.6 ± 4.4	26.5 ± 4.7	0.007*
z score, median (IQR)	2.8 (2.6)	2.5 (2.2)	0.251

Data are presented in mean ± SD or *n* (%), unless otherwise indicated

*p-values < 0.05 were considered statistically significant

**Table 4:** Sinus of Valsalva and ascending aortic diameter changes from baseline in the two groups during the 2 years follow-up

Clinical features	Physically active subjects' group (*n* = 53)	Sedentary subjects' group (*n* = 37)	*p*-value
Sinuses of Valsalva diameter, changes from baseline (mm)	0.1 ± 2.2	0.7 ± 1.2	0.098
z score, median (IQR)	0.0 (0.3)	0.1 (0.3)	0.104
Ascending aorta diameter, changes from baseline mm	0.1 ± 2.0	0.9 ± 2.0	0.056
z score, median (IQR)	0.0 (0.6)	0.0 (0.5)	0.174

Data are presented in mean ± SD or *n* (%), unless otherwise indicated

*p-values < 0.05 were considered statistically significant

Compared to sedentary, physically active subjects showed no significant difference regarding the rate of sinus of Valsalva diameter (13% vs. 8%, *p*-value = 0.449) and ascending aorta diameter progression (9% vs. 13%, *p*-value = 0.545) during the 2 years follow-up. Moreover, no significant difference was observed in the degree of dilation between physically active and sedentary subjects at 2 years follow-up, either in subjects with sinus of Valsalva diameter progression (3.7 ± 1.0 mm *vs*. 3.5 ± 0.8 mm, *p*-value = 0.67) (Table 5), or in those with ascending aorta diameter progression (3.0 ± 0.8 mm *vs*. 3.2 ± 1.3 mm, *p*-value = 0.83) (Table 6).

**Table 5:** Sinus of Valsalva diameter changes from baseline in subjects with sinus of Valsalva diameter progression during the 2 years follow-up

Clinical features	Physically active subjects' group (*n* = 7)	Sedentary subjects' group (*n* = 3)	*p*-value
Sinuses of Valsalva diameter, changes from baseline mm	3.7 ± 1.0	3.5 ± 0.8	0.673
z score, median (IQR)	1.0 (0.2)	1.0 (0.2)	0.747

Data are presented in mean ± SD or *n* (%), unless otherwise indicated

*p-values < 0.05 were considered statistically significant

**Table 6:** Ascending aorta diameter changes from baseline in subjects with ascending aorta diameter progression during the 2 years follow-up

Clinical features	Physically active subjects' group (*n* = 5)	Sedentary subjects' group (*n* = 5)	*p*-value
Ascending aorta diameter, changes from baseline mm	3.0 ± 0.8	3.2 ± 1.3	0.830
z score, median (IQR)	0.8 (0.6)	0.8 (0.5)	0.593

Data are presented in mean ± SD or *n* (%), unless otherwise indicated

*p-values < 0.05 were considered statistically significant

In patients with sinus of Valsalva dilation at baseline, no significant difference was observed in the aortic diameter changes between physically active and sedentary subjects at 2 years follow-up (1.6 ± 1.6 mm *vs*. 0.0 ± 2.9 mm, *p*-value = 0.216) (Table 7). Similarly, in patients with ascending aorta dilation at baseline, no significant difference was observed in the aortic diameter changes between physically active and sedentary subjects at 2 years follow-up (1.4 ± 1.6 mm *vs*. 0.4 ± 1.3 mm, *p*-value = 0.957) (Table 8).

**Table 7:** Sinus of Valsalva diameter changes from baseline in subjects with sinus of Valsalva dilation at baseline during the 2 years follow-up

Clinical features	Physically active subjects' group (*n* = 8)	Sedentary subjects' group (*n* = 5)	*p*-value
Sinuses of Valsalva diameter, changes from baseline mm	1.6 ± 1.6	0.0 ± 2.9	0.216
z score, median (IQR)	0.3 (0.9)	-0.1 (0.9)	0.061

Data are presented in mean ± SD or *n* (%), unless otherwise indicated

*p-values < 0.05 were considered statistically significant

**Table 8:** Ascending aorta diameter changes from baseline in subjects with ascending aorta dilation during the 2 years follow-up

Clinical features	Physically active subjects' group (*n* = 33)	Sedentary subjects' group (*n* = 21)	*p*-value
Ascending aorta diameter, changes from baseline mm	1.4 ± 1.6	1.4 ± 1.3	0.957
z score, median (IQR)	0.2 (0.6)	0.3 (0.5)	0.906

Data are presented in mean ± SD or *n* (%), unless otherwise indicated

*p-values < 0.05 were considered statistically significant

## Discussion

Aortic dissection or rupture of an underlying congenital or inherited aortic dilatation/aneurism is an important cause of sudden cardiac death (SCD) in athletes [15]. Bicuspid aortic valve (BAV) patients have an increased incidence of developing aortic dilation that can involve the aortic root and/or the ascending aorta, potentially leading to an aortic aneurysm [16–23]. Several theories have been proposed to explain the pathogenesis of aortopathy in these patients [24], and it is supposed that genetic or epigenetic variation and environmental modifiers can cause BAV-associated aortopathy [25, 26]. It is a belief that increased blood pressure and aortic wall shear stress during physical exertion may increase the risk of aneurysm formation, aortic dissection or rupture in patients with genetic syndrome associated with aortopathies [10], but the role of regular physical training in BAV patients has not been fully evaluated.

Recently, Boraita et al. [27] studied the prevalence and the characteristics of BAV among elite athletes to analyse the effect of long-term exercise training on their aortas. Of 5316 elite athletes, 41 subjects with BAV were identified and, among these, 16 athletes had undergone two or more cardiac evaluations to assess their clinical course. In their small cohort, no significant difference in aortic diameter or valve function was found during 7 years follow-up, suggesting that high intensity training and sport competition may not trigger aortic enlargement or aortic valve dysfunction among BAV subjects during their athletic careers.

In this study, we evaluated the impact of regular physical activity on aortic diameters (sinus of Valsalva and ascending aorta) in a consecutive cohort of paediatric patients with BAV, categorized in two groups: physically active and sedentary subjects. We observed no association between exercise and aortic diameter progression during 2-year follow-up. Similarly, in subjects with aortic dilation observed during follow-up, there was no significant difference in dilation between the two groups. Thus, our data suggest that aortic root and ascending aorta dilation are not associated with regular physical activity in paediatric BAV patients during 2-year follow-up.

The recommendations for sports eligibility for patients with BAV are consistent with the ACC/AHA valve and aorta guidelines [10, 27], and should be tailored to anatomical concern and sport demands. In these subjects, the risk of SCD coming from aortic rupture or dissection is related most commonly to progressive valvular heart disease and aorta dilation [6]. Accumulating evidence that regular sport does not influence the aortic diameter progression in BAV patients may be of high relevance in clinical practice. These finding may minimize the inappropriate disqualification of BAV patients from sport, in particular in children and adults with mild aortic disease.

### Limitations

Our study has several limitations, among others: small sample size; echo measurements of aortic root and mid-ascending aorta performed in a single plane by different echocardiographists (interobserver variability); various methods of measurement of the aortic root and of the ascending aorta (L-L convention in the present study, inner edge to inner edge in the study used for the Z score calculation [14]). Future multicentric study based on larger populations are needed to confirm these results.

## Conclusions

In our paediatric cohort of BAV patients, the prevalence and the degree of aortic diameter progression was not significantly different between physically active and sedentary subjects at 2 years follow-up, suggesting that further aortic dilation is not related to regular physical activity.

## Appendix 1: List of BAVCon sites

**Table:**

Site	Principal investigator	Email
Brigham and Women’s Hospital	Simon C. Body	scbody@bu.edu
Laval	Yohan Bossé	Yohan.Bosse@criucpq.ulaval.ca
Mayo	Hector I. Michelena	michelena.hector@mayo.edu
Massachusetts General Hospital	Thoralf M. Sundt	tsundt@mgh.harvard.edu
Michigan	Bo Yang	boya@med.umich.edu
Oxford	Malenka Bissell	malenka.bissell@cardiov.ox.ac.uk
San Donato (Milan)	Francesca Pluchinotta	francesca.pluchinotta@grupposandonato.it
University of Texas, Houston	Dianna M. Milewicz	dianna.M.Milewicz@uth.tmc.edu
Tufts University	Gordon Huggins	ghuggins@tuftsmedicalcenter.org
Vall d’Hebron (Barcelona)	Arturo Evangelista	arturevangelistamasip@ gmail.com
Vanderbilt	Joshua C. Denny	josh.denny@vanderbilt.edu
